# 2138. *In Vitro* Susceptibility of Recent Clinical Isolates of *P. aeruginosa* and *Enterobacterales* to Imipenem or Meropenem Alone or in Combination with Sulbactam-Durlobactam

**DOI:** 10.1093/ofid/ofad500.1761

**Published:** 2023-11-27

**Authors:** Sarah McLeod, Nicole Carter, Samir Moussa, Alita Miller

**Affiliations:** Innoviva Specialty Therapeutics, Waltham, Massachusetts; Innoviva Specialty Therapeutics, Waltham, Massachusetts; Entasis Therapeutics, Waltham, Massachusetts; Entasis Therapeutics, Waltham, Massachusetts

## Abstract

**Background:**

Sulbactam-durlobactam (SUL-DUR) is a targeted β-lactam/β-lactamase inhibitor combination in development for the treatment of infections caused by *Acinetobacter* spp., including multidrug resistant strains. ATTACK was a global, active-controlled Phase 3 trial conducted to evaluate the efficacy and safety of SUL-DUR vs. colistin for patients with *Acinetobacter* infections. Both arms were dosed on a background of imipenem/cilastatin to treat co-infecting Gram-negative pathogens. Here, the in vitro activity of carbapenems with or without SUL-DUR added against *P. aeruginosa* (PA) or *Enterobacterales* is presented.

**Methods:**

Broth minimal inhibitory concentrations (MICs) for single, double, and triple combinations of sulbactam, durlobactam and imipenem or meropenem were determined following CLSI methodologies. Two different sets of isolates were tested: 104 molecularly characterized clinical isolates and 69 co-infecting baseline pathogens from the ATTACK trial.

**Results:**

Against 55 *Enterobacterales* isolates, the MIC_90_s of imipenem and meropenem were reduced from 32 and >32 μg/ml to 2 μg/ml in the presence of durlobactam. Against 49 carbapenem-resistant *P. aeruginosa*, addition of durlobactam reduced the imipenem MIC_90_ 8-fold from 32 to 4 μg/ml and the meropenem MIC_90_ from 64 to 16 μg/ml. Of the 39 co-infecting pathogens from ATTACK, 30 (43%) were carbapenem non-susceptible (8 PA, 21 *Klebsiella pneumoniae* and 1 *Proteus mirabilis*). Addition of durlobactam restored imipenem susceptibility to 83% (N = 25) and meropenem susceptibility to 70% (N = 21) of these isolates. No antagonism was observed for any durlobactam combination tested. The addition of sulbactam had no effect on the antibacterial activity of either carbapenem -/+ durlobactam added for any of the isolates in this study.

**Conclusion:**

The addition of durlobactam restored the *in vitro* antibacterial activity of imipenem and meropenem against a majority of carbapenem-resistant *Enterobacterales* and *P. aeruginosa* clinical isolates, while the addition of sulbactam had no effect on any combination tested. These results indicate that durlobactam has the potential to restore carbapenem activity against these organisms when present in *Acinetobacter* polymicrobial infections.

**Disclosures:**

**Sarah McLeod, PhD**, Innoviva Specialty Therapeutics: Employee|Innoviva Specialty Therapeutics: Employee|Innoviva Specialty Therapeutics: Stocks/Bonds|Innoviva Specialty Therapeutics: Stocks/Bonds **Nicole Carter, MS**, Innoviva Specialty Therapeutics: Employee|Innoviva Specialty Therapeutics: Stocks/Bonds **Samir Moussa, PhD**, Innoviva: Stocks/Bonds|Innoviva Specialty Therapeutics: Employee|Innoviva Specialty Therapeutics: Stocks/Bonds **Alita Miller, PhD**, Entasis Therapeutics: employee|Entasis Therapeutics: Stocks/Bonds

